# Supplementary material for: The interactome of intact mitochondria by cross-linking mass spectrometry provides evidence for coexisting respiratory supercomplexes
Source: Mol Cell Proteomics. 2017 Dec 8;17(2):216–32. doi: 10.1074/mcp.RA117.000470 (PMC5795388; doi:10.1074/mcp.RA117.000470)
Supplement: Supplemental Data [file supp_RA117.000470_133922_0_supp_23978_fzffwf.zip › spectra_annotation/mito_spectra_annotation/394-1-8-1-8-1.pdf]

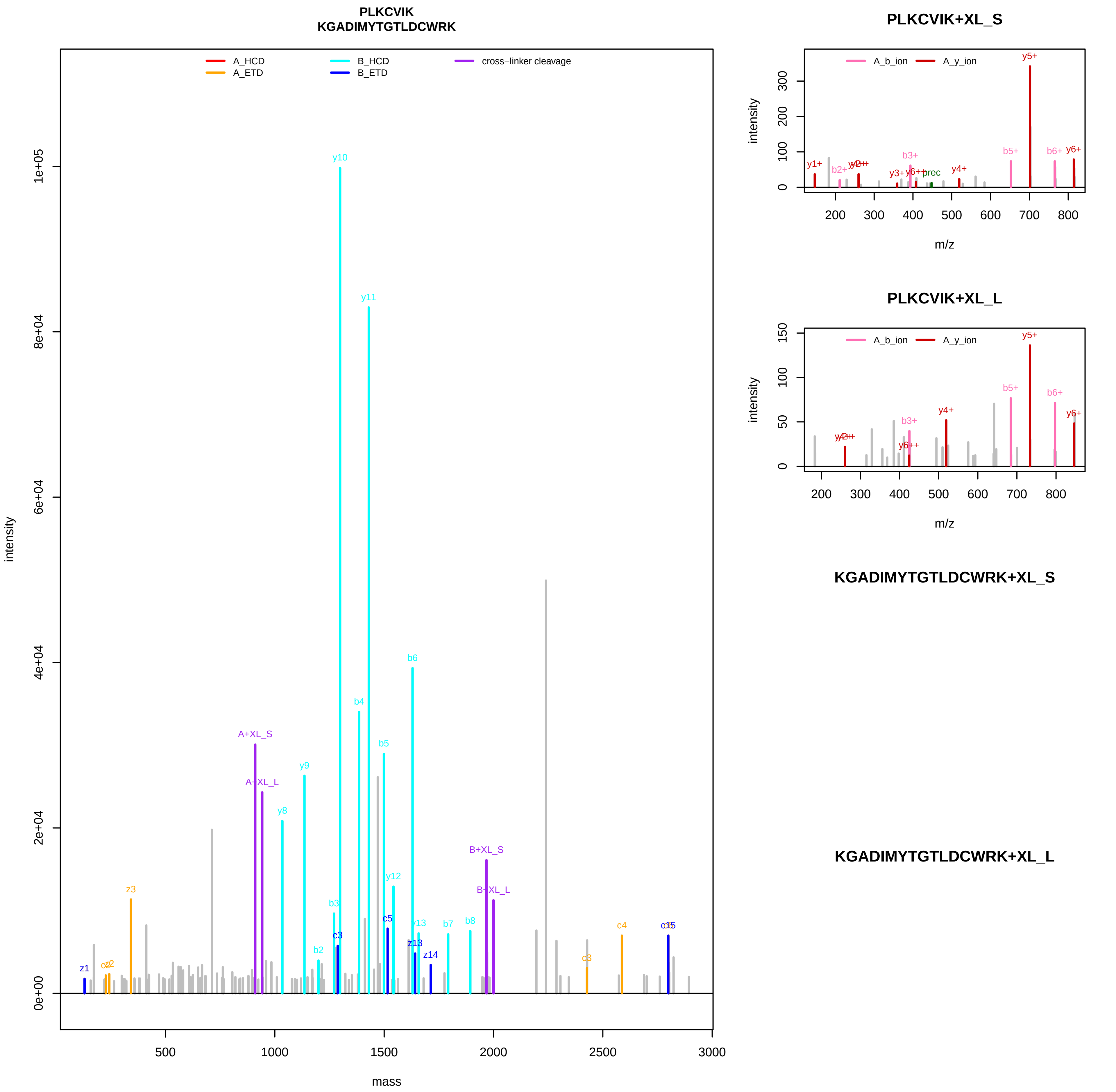
PLKCVIK
KGADIMYTGTLDCWRK
A_HCD
A_ETD
B_HCD
B_ETD
cross−linker cleavage
intensity
mass
0e+00
2e+04
4e+04
6e+04
8e+04
1e+05
500
1000
1500
2000
2500
3000
y10
y11
b6
b4
b5
A+XL_S
A+XL_L
y9
y8
B+XL_S
B+XL_L
y12
z3
b3
c5
c3
b2
y13
b7
b8
z13
z14
c4
c3
c15
z1
PLKCVIK+XL_S
A_b_ion
A_y_ion
y5+
b5+
b6+
y6+
b3+
y4+
prec
y1+
b2+
y3+
y6++
m/z
PLKCVIK+XL_L
KGADIMYTGTLDCWRK+XL_S
KGADIMYTGTLDCWRK+XL_L
